# Comparative toxicogenomics of three insensitive munitions constituents 2,4-dinitroanisole, nitroguanidine and nitrotriazolone in the soil nematode *Caenorhabditis elegans*

**DOI:** 10.1186/s12918-018-0636-0

**Published:** 2018-12-14

**Authors:** Ping Gong, Keri B. Donohue, Anne M. Mayo, Yuping Wang, Huixiao Hong, Mitchell S. Wilbanks, Natalie D. Barker, Xin Guan, Kurt A. Gust

**Affiliations:** 1 Environmental Laboratory, U.S. Army Engineer Research and Development Center, 3909 Halls Ferry Road, Vicksburg, MS 39180 USA; 2 Division of Bioinformatics and Biostatistics, National Center for Toxicological Research, U.S. Food and Drug Administration, Jefferson, AR 72079 USA; 3 Bennett Aerospace Inc., 1249 Kildaire Farm Road, Cary, NC 27511 USA

**Keywords:** Toxicogenomics, Acute toxicity, *Caenorhabditis elegans*, Insensitive munitions, Microarray, Differentially expressed genes, Gene set enrichment analysis (GSEA), Kyoto Encyclopedia of Genes and Genomes (KEGG) pathways

## Abstract

**Background:**

Ecotoxicological studies on the insensitive munitions formulation IMX-101 and its components 2,4-dinitroanisole (DNAN), nitroguanidine (NQ) and nitrotriazolone (NTO) in various organisms showed that DNAN was the main contributor to the overall toxicity of IMX-101 and suggested that the three compounds acted independently. These results motivated this toxicogenomics study to discern toxicological mechanisms for these compounds at the molecular level.

**Methods:**

Here we used the soil nematode *Caenorhabditis elegans*, a well-characterized genomics model, as the test organism and a species-specific, transcriptome-wide 44 K-oligo probe microarray for gene expression analysis. In addition to the control treatment, *C. elegans* were exposed for 24 h to 6 concentrations of DNAN (1.95–62.5 ppm) or NQ (83–2667 ppm) or 5 concentrations of NTO (187–3000 ppm) with ten replicates per treatment. The nematodes were transferred to a clean environment after exposure. Reproduction endpoints (egg and larvae counts) were measured at three time points (i.e., 24-, 48- and 72-h). Gene expression profiling was performed immediately after 24-h exposure to each chemical at the lowest, medium and highest concentrations plus the control with four replicates per treatment.

**Results:**

Statistical analyses indicated that chemical treatment did not significantly affect nematode reproduction but did induce 2175, 378, and 118 differentially expressed genes (DEGs) in NQ-, DNAN-, and NTO-treated nematodes, respectively. Bioinformatic analysis indicated that the three compounds shared both DEGs and DEG-mapped Reactome pathways. Gene set enrichment analysis further demonstrated that DNAN and NTO significantly altered 12 and 6 KEGG pathways, separately, with three pathways in common. NTO mainly affected carbohydrate, amino acid and xenobiotics metabolism while DNAN disrupted protein processing, ABC transporters and several signal transduction pathways. NQ-induced DEGs were mapped to a wide variety of metabolism, cell cycle, immune system and extracellular matrix organization pathways.

**Conclusion:**

Despite the absence of significant effects on apical reproduction endpoints, DNAN, NTO and NQ caused significant alterations in gene expression and pathways at 1.95 ppm, 187 ppm and 83 ppm, respectively. This study provided supporting evidence that the three chemicals may exert independent toxicity by acting on distinct molecular targets and pathways.

**Electronic supplementary material:**

The online version of this article (10.1186/s12918-018-0636-0) contains supplementary material, which is available to authorized users.

## Background

The history of insensitive munitions (IMs) can be traced back to June 1978 when the U.S. Department of Defense (DoD) and Department of Energy (DOE) agreed to undertake a joint effort to study the utility of insensitive high explosives and propellants in some typical DoD conventional weapons systems [1, 2]. Unlike conventional ordnance, IMs are not prone to detonate due to shock, heat or fire, and hence enhances the safety of occupational workers and soldiers during manufacturing, transportation, storage, and use for testing, training and military operations [2]. In 2010, the U.S. Army approved IMX-101 (an acronym for Insensitive Munitions Explosive 101) as the first IM formulation, which passed all six criteria, i.e., fragment impact, shaped charge jet impact, slow cook-off, fast cook-off, multiple bullets into high explosives, and sympathetic detonation [3].

**Fig. 1:**
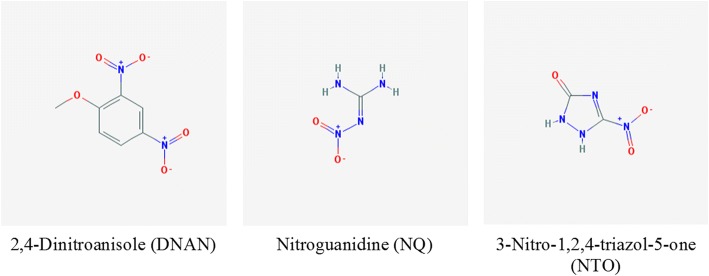
The 2D structures of three IMX-101 constituents

To fully characterize the potential human health and environmental risks associated with exposure to IMX-101, many toxicological and ecotoxicological studies have been conducted with IMX-101 and its three constituents: 2,4-dinitroanisole (DNAN, replacing 2,4,6-trinitrotoluene or TNT), 3-Nitro-1,2,4-Triazol-5-One (NTO, replacing 1,3,5-trinitro-1,3,5-triazacyclohexane also known as Research Department formula X or RDX), and nitroguanidine (NQ) (see Fig. 1 for their chemical structures). In vivo (including acute, subacute, and subchronic exposures) and in vitro toxicity testing results suggest that DNAN [4] has moderate toxicity whereas NQ [5] and NTO [6] have no or low toxicity to a wide variety of physiological, histopathological, reproductive, developmental, genotoxicity, and mutagenicity endpoints in mammalian rodents such as rats, mice and rabbits. In a 14-day subacute toxicity study, DNAN caused anemia and hepatocellular injury in female rats and hyperalbuminemia in male rats [7]. In another 90-day subchronic study, oral gavage of DNAN led to mortality at the highest dose (80 mg/kg/day), and other sublethal effects including altered neuromuscular functions (neurotoxicity), anemia, splenic enlargement, hemosiderosis and extramedullary hematopoiesis (all of which indicated blood as a target organ), as well as testicular toxicity manifested as decreased mass of testes and epididymides, seminiferous tubule degeneration and epididymal aspermia in males [7]. A single administration of 120 and 150 mg DNAN/kg caused mortality in 2-week old male Japanese quails (1/5 and 5/9 dosed quails, respectively) and cataracts in all of the surviving quails [8]. Another study suggests that DNAN can induce maternal reproductive toxicity, embryo toxicity and fetus teratogenicity in pregnant rats dosed with 5, 15 and 45 mg/kg/d for 2 weeks during gestation [9]. The primary effect of concern for NTO is testicular toxicity observed in rats and mice in both subchronic and extended one-generation reproductive toxicity tests [10–13]. NTO does not exhibit estrogenic or antiandrogenic endocrine disrupting [14] or neurobehavioral [10] effects in rats at doses up to 1000 mg/kg/d. Reddy et al. [15] revealed that NTO was not genotoxic using a battery of in vitro and in vivo genotoxicity tests. The low toxicity of NTO may be attributed to its low electron-accepting property as demonstrated by its reaction with both single-electron and two-electron transferring flavoenzymes [16].

Ecotoxicological assessment of the three IM constituents has been mostly performed in aquatic organisms. DNAN severely inhibited methanogens, nitrifying bacteria, and *Aliivibrio fischeri* with 50% inhibitory concentrations (IC_50_) ranging 41–57 μM but was notably less inhibitory to aerobic heterotrophs (IC_50_ > 390 μM) [17]. Dodard et al. [18] observed the following ecotoxicity for DNAN: 50% effect concentrations (EC_50_) of 4.0 mg/L for 72-h *Pseudokirchneriella subcapitata* (green alga) growth, 7 mg/kg for 19-d *Lolium perenne* (ryegrass) growth, 60.3 mg/L for 30-min *A. fischeri* bacteria bioluminescence, and 31 mg/kg for 48-h *Eisenia andrei* (earthworm) soil avoidance, and a 14-d 50% lethal concentration (LC_50_) of 47 mg/kg for *E. andrei*. DNAN induced DNA damages in *Daphnia carinata* with a 48-h LC_50_ of 15 mg/L derived from a comet assay [19]. Kennedy et al. [20] reported acute and chronic toxicity of DNAN to *Pimephales promelas* (fathead minnow) and two cladocerans (*Ceriodaphnia dubia* and *Daphnia pulex*): acute LC_50_ ranging from 14.2 mg/L to 42 mg/L, chronic LC_50_ 10 mg/L to > 24.2 mg/L, and reproductive IC_50_ 2.7 mg/L to 10.6 mg/L, with *D. pulex* being the most sensitive species. Using *Rana pipiens* (leopard frog) tadpoles as the test organism, Stanley et al. [21] observed a 96-h LC_50_ of 24.3 mg/L for DNAN and a 28-d mortality LOEC (lowest observable effect concentration) of 2.4 mg/L and 5.0 mg/L for DNAN and NTO, respectively. However, neither tadpole developmental stage nor growth was significantly affected in any of the 28-d exposures [21]. NQ has low acute toxicity to fish including rainbow trout (*Ongorhynchus myisi*), fathead minnows, channel catfish (*Ictalurus punctatus*), and bluegills (*Lepomis macrochirus*), invertebrates including water fleas (*Daphnia magna*), amphipods (*Hyallela azteca* and *Gammarus minus*), midge larvae (*Paratanytarsus dissimilis*), and aquatic worms (*Lumbriculus variegatus*), and algae (*Selenastrum capricornutum*) up to the water solubility limit level [22]. Chronic toxicity data of NQ show the non-observable adverse effect level (NOAEL) 260 mg/L and lowest observable adverse effect level (LOAEL) 440 mg/L in *C. dubia* [23], the LOEC 2030 mg/L and no observable effect concentration (NOEC) 1050 mg/L (based on early life stage reduction in total length) in fathead minnows [23], and not toxic to rainbow trout up to saturation [22, 23].

Other data from ongoing investigations in our laboratory suggest that DNAN, NTO and NQ elicited independent toxicity to test organisms including fathead minnow larvae [24], the fresh-water amphipod *Hyallela azteca* (Lotufo et al. Unpublished data) and the earthworm *Eisenia fetida* (Gong et al. Unpublished data). In agreement with published results, our data also indicated that DNAN and NTO account for the majority of toxicity exerted by IMX-101 with DNAN being more toxic than NTO. Nevertheless, mode-of-action results for the three IM constituents are only now beginning to emerge, e.g., in fish species [24, 25], but a significant knowledge gap remains. In order to fill this knowledge gap, we launched the present toxicogenomics study to investigate the toxicological mechanisms of IM constituents, where we hypothesized that the three chemicals would act independently on different molecular targets and affect different biological pathways in *Caenorhabditis elegans*. *C. elegans* was chosen as the test organism because toxicity testing in this organism can bridge genetic, biochemical, developmental and physiological endpoints [26]. In addition, *C. elegans* is a free-living organism with a small (1 mm in length) and transparent body, a fully described developmental program, and a short life cycle [27]. Of importance for genomics studies, it has a completely sequenced (ca. 100 Mb) and well-annotated (20,362 protein coding and 24,719 non-coding genes) genome ([28]; also see http://useast.ensembl.org/Caenorhabditis_elegans/Info/Annotation). There is increasing evidence that toxicity testing results obtained using *C. elegans* are predictive of outcomes in higher eukaryotes including humans, likely due to genetic conservation and physiological similarity [29]. For instance, 90% of human lysosome associated non-disease genes and 70% of human lysosomal storage disorder genes have *C. elegans* homologs [30], allowing for rapid screening of lysosomal toxins using *C. elegans* in vivo assays [31]. In addition, good correlation was reported for metal salts between *C. elegans* lethality ranking and mammalian oral LD_50_ ranking [32, 33]. All these features make *C. elegans* an excellent toxicogenomics model for identification of molecular targets as well as rapid risk assessment of uncharacterized chemicals [26, 29] including IM compounds.

## Methods

### Animal culturing and preparation

The wild-type *C. elegans* var. Bristol, strain N2, was maintained at 20 °C on NGM (Nematode Growth Media) agar plates seeded with *Escherichia coli* OP50 bacteria according to standard protocols [34, 35]. The culture was synchronized at the first larval stage (L1) by bleaching with a 5% sodium hypochlorite solution [35, 36]. Worms were harvested by gentle rinsing with K Medium in late L3 stage which were determined by both length and age [27, 37]. Worms of larval stage L3 were used in 24-h acute exposures to ensure that worms would not reach the egg-laying adult age within the time period of exposure, thus eliminating the confounding effect of RNA from laid or unlaid eggs being incorporated into genomic analysis. After harvesting from agar plates, worms were spun at 1150×g for 2.5 min and re-suspended in a given volume of K Medium [36]. Twenty μL of worms were deposited on a microscope slide with a grid, and all the worms in the grid were counted. The number of worms/mL was then calculated. Wide-bore pipet tips or transfer pipets were always used to avoid shearing of worms.

### Acute exposure

Worms in late L3 stage were used for acute toxicity testing [37]. Three sets of experiments were performed: viability, reproduction and gene expression, where worms were exposed in tissue-culture treated (TC) plates for 24 h to DNAN, NTO, or NQ [38]. All test solutions were prepared in K medium except that acetone was used to dissolve DNAN, resulting in 5% carrier in the final solution. In addition to the vehicle or blank controls, the viability assay tested at least 5 concentrations per compound with 3 replicates per concentration. The nominal concentrations in viability assays were: vehicle control (VC), 1.95, 3.91, 7.81, 15.6, 31.2, 62.5, 125, 250, 500 and 1000 mg DNAN/L, blank control (BC), 187.5, 375, 750, 1500 and 3000 mg NTO/L, and BC, 93.75, 187.5, 375, 750 and 1500 mg NQ/L. Those in reproduction assays were: VC, 1.95, 3.91, 7.81, 15.6, 31.2, 62.5 mg DNAN/L, BC, 187.5, 375, 750, 1500, 3000 mg NTO/L, and BC, 83, 167, 333, 666, 1333, 2667 mg NQ/L. Those in the genomics study were: VC, 1.9, 15.5, 62 mg DNAN/L, BC, 187, 750, 3000 mg NTO/L, and BC, 83, 666, 2667 mg NQ/L. For viability and reproduction studies, 96-well TC plates were used with ca. 30 worms housed in a well and each well was filled with 100 μl of K medium or chemical-amended solution. For gene expression studies, 24-well TC plates were used with 400 worms per well and each well was filled with 1 ml of K medium or test solution. Each treatment in the reproduction and genomics studies had 5 replicates.

### Viability and reproduction experiments

In both experiments, prior to and immediately after exposure, dead and live worms in each well were confirmed and counted. After exposure, six surviving worms from each well in the reproduction study (30 worms in total) were transferred to an agar plate for rinsing to remove test chemical. Then, each rinsed worm was placed in a new well containing 100 μl of K medium and *E. coli* OP50 bacteria at an optical density (OD) of 0.2, and allowed to reproduce. At 24, 48 and 72 h, plates were fixed with a 5% final volume of paraformaldehyde and stored at 4 °C. At each time point, 10 wells per treatment were examined. Offspring, including laid eggs and larvae, were counted and any mortality of adults was also recorded [37]. Statistical analysis of viability and reproduction data (i.e., analysis of variance or ANOVA) was performed using the AOV function in R (version 3.3.1).

### Microarray design

Global gene expression was analyzed using a custom-designed Agilent *C. elegans*-specific microarray that contained 42,862 probes targeting 28,164 unique genes, among which 76.6% or 21,562 genes had a single probe, 23.4% or 6596 genes had 2 to 12 probes, and the remaining 6 genes had 13, 15, 20, 26, 27 and 37 probes, respectively. These probes were assembled from three existing Agilent probe groups: PGRID51529TDT (512 probes), PGRID51764TDT (50), and PGRID51763TDT (42300). These 60-mer oligo probes, together with 1319 control probes, were printed in situ on a 364-row by 164-column array grid in an 8-array-per-slide format, yielding a 70% occupancy of 62,976 available spots. This custom-commercial hybrid array was deposited in the Gene Expression Omnibus (GEO) repository as platform GPL22795 (https://www.ncbi.nlm.nih.gov/geo/query/acc.cgi?acc=GPL22795).

### Microarray experiment

After the 24-h exposure, all 400 worms per well were collected into a cryogenic vial and spun at 1150×g for 2.5 min. Supernatant was removed and the vial were flash frozen in liquid nitrogen and stored at − 80 °C. Total RNA was extracted from each vial using RNeasy mini kits (Qiagen, Valencia, CA) and treated as one biological replicate. Four of the five replicates (total RNA samples) per treatment were chosen for further microarray hybridization. One hundred ng of total RNA was first reverse-transcribed into cDNA, followed by cDNA labeling using Low Input Quick Amp Labeling Kits (Agilent, Palo Alto, CA) in the presence of cyanine 3-CTP dye. The labeled cDNA was hybridized to the *C. elegans*-specific 44 K-olig array (one sample/array) at 65 °C for 17 h using Agilent’s Gene Expression Hybridization Kits [39]. A total of 48 samples (3 chemicals × 4 treatments per chemical × 4 replicates per treatment) were randomly assigned and hybridized to six 8 × 60 K-array slides containing 44 K oligonucleotide probes per array.

### Gene expression data analysis

An Agilent high-resolution DNA Microarray Scanner Model G2565CA was used to scan microarray images (resolution = 2 μm per pixel). Raw microarray gene expression data were extracted from acquired array images as spot and background signal intensity using Agilent’s Feature Extraction software v10.7. A spot was flagged out if its raw signal intensity was below its background level, or if it was saturated. A gene was flagged out if over 50% of the 48 samples had missing values for this gene. The filtered data was background subtracted and log-transformed. The spike-in RNA mix with known composition and RNA copy numbers was used to construct standard linear regression curves, from which the unknown worm RNA concentrations were derived. The derived RNA concentrations were normalized to the median value per array (sample). The raw and normalized array data were deposited in the GEO database as series GSE92365 (https://www.ncbi.nlm.nih.gov/geo/query/acc.cgi?acc=GSE92365). Multivariate permutation tests with random variance model implemented in BRB-Array Tools version 4.5 [40] were performed to infer differentially expressed genes (DEGs). One thousand random permutations were computed per chemical class (i.e., a group of 16 arrays or samples). The confidence level of false discovery rate assessment was set at 80%, and the maximum allowed portion of false-positive genes was 10%.

### Pathway analysis

Pathway mapping was conducted to map DEGs to computationally inferred *C. elegans* pathways in the Reactome Knowledgebase Version 56 (www.reactome.org; [41]). Gene set enrichment analysis (GSEA) [42] was performed to derive KEGG (Kyoto Encyclopedia of Genes and Genomes) pathways (www.genome.jp/kegg/; [43]) statistically enriched with DEGs. A GSEA v3.0 beta 2 desktop application was downloaded from http://software.broadinstitute.org/gsea/downloads.jsp.

Reactome and KEGG contain both manually curated and computationally inferred biological pathways which are updated regularly. For example, KEGG releases 1–2 new pathway maps every month and constantly updates existing maps [43]. In the current study, we downloaded the V56 Reactome and the then-most-updated KEGG pathways released in or before June 2016. There were 134 KEGG pathways and 1434 Reactome pathways computationally inferred for *C. elegans* from their orthologous reference pathways in model organisms such as humans, mice, rats and fruit flies, which were manually curated. Hence, the findings reported here should be regarded as those derived and interpreted using the best knowledge available at the time of the study.

Pathways in Reactome are organized hierarchically, following the gene ontology (GO) biological process hierarchy [41]. At the highest hierarchical level, there are 26 groups of pathways such as cell cycle, cell-cell communication, developmental biology, DNA repair and replication, DNA and protein metabolism, immune system, muscle contraction, reproduction and signal transduction. Unlike Reactome, KEGG assigns genes molecular level functions defined in > 20,000 KEGG Orthology (KO) entries, which are mapped to six categories of manually drawn regular KEGG reference pathway maps, including 160 metabolism, 22 genetic information processing, 38 environmental information processing, 24 cellular processes, 78 organismal systems and 81 human diseases pathways [43].

## Results

### Acute toxicity of DNAN, NTO and NQ to *C. elegans*

**Fig. 2:**
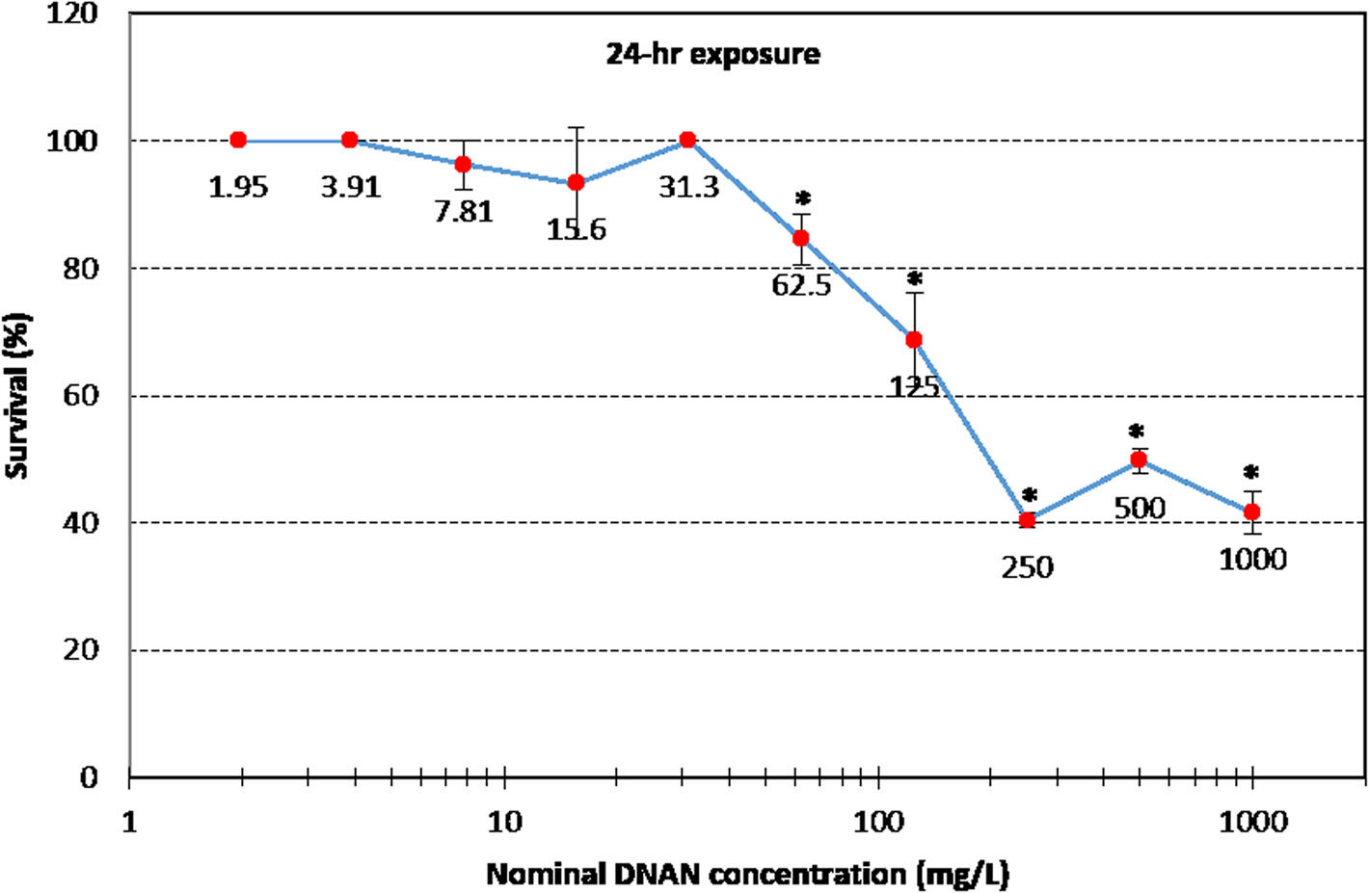
Effects of 24-h exposure to DNAN on *C. elegans* viability (*n* = 3 with each replicate having ca. 30 worms, error bar = standard deviation). “*” represents statistical significance between treatment and control at *p* < 0.01 (ANOVA with Tukey’s honest significance test)

**Fig. 3:**
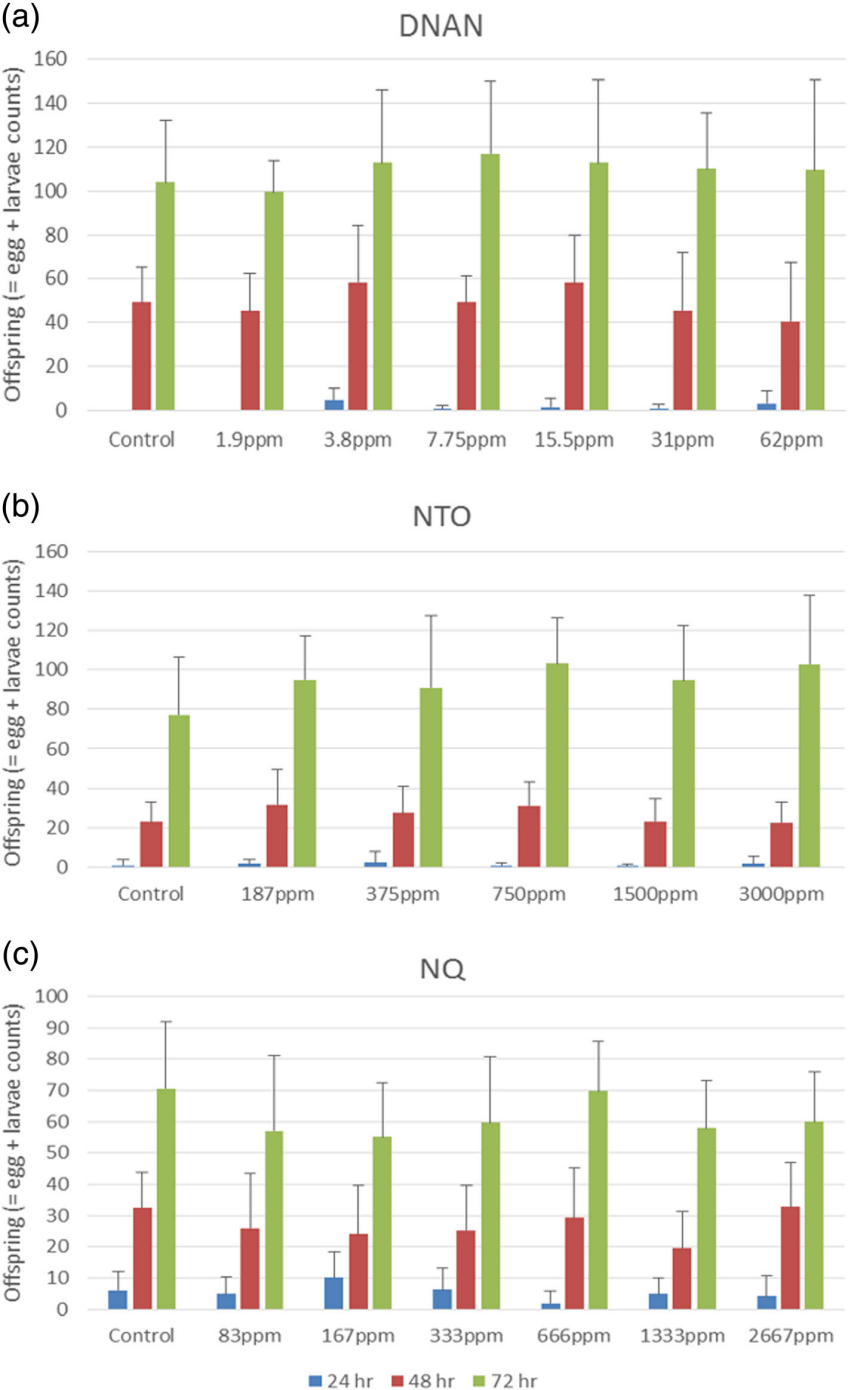
Offspring produced per single *C. elegans* adult after 24-h exposure to (**a**) DNAN, (**b**) NTO or (**c**) NQ at 24-, 48- and 72-h time points post-exposure (*n* = 7–10 where each replicate followed reproduction of single individuals, error bar = standard deviation). There were no significant differences observed in response to the chemical exposures (*p* > 0.05, ANOVA)

No statistically significant effects of NTO and NQ were observed on the survival of L3-stage worms in the acute 24-h exposures (data not shown). However, DNAN caused significant mortality (*p* < 0.01, ANOVA with Tukey’s honest significance test) reducing survivorship by nearly 60% in the 250 mg/L exposure (Fig. 2) with a LOEC of 62.5 mg DNAN/L in the *C. elegans* acute exposure. Regarding the reproduction endpoints, acute exposure to the three compounds at non-lethal concentrations, i.e., 1.9–62.5 mg DNAN/L, 187–3000 mg NTO/L, or 83–2667 mg NQ/L, did not significantly affect offspring production (i.e., egg or larvae counts and the sum of both) at any of the three time points, i.e., 24-, 48-, and 72-h post exposure (Fig. 3). Statistical comparisons of the controls for the DNAN, NTO and NQ experiments showed no significant difference (*p* > 0.05, ANOVA) at each of the three time points, demonstrating consistent performance of the *C. elegans* across the three toxicity tests. The reason why the 62.5 mg DNAN/L treatment caused mortality but no reproductive toxicity was likely due to the fact that only surviving worm were used in the reproductive assays. These acute toxicity testing results are consistent with reported toxicity for these compounds in other organisms.

### Sublethal transcriptional effects

**Fig. 4:**
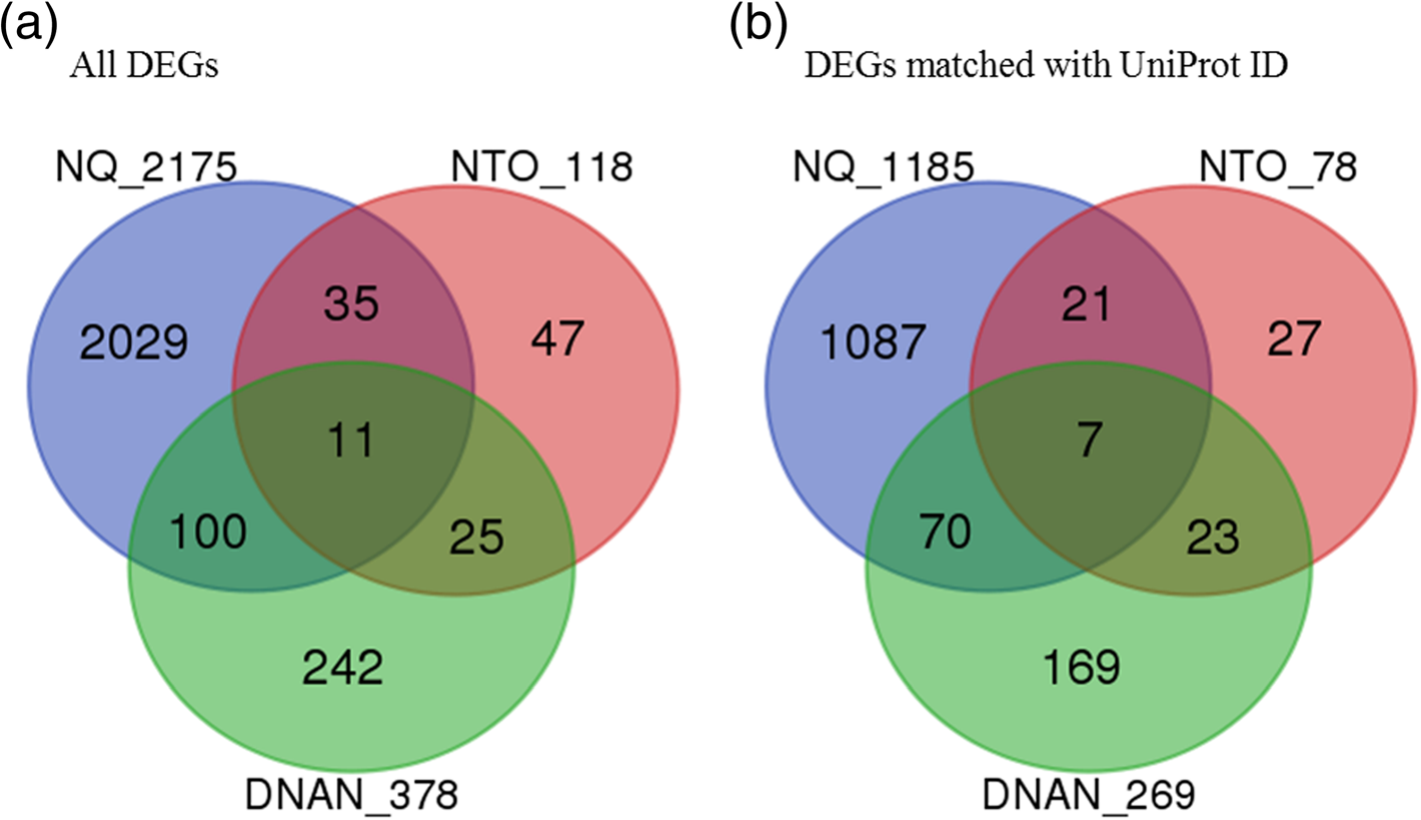
Overlapping of (**a**) differentially expressed genes (DEGs) inferred from DNAN, NTO, and NQ treatments as well as (**b**) of those DEGs matched with a UniProt identifier

Initial filtering of the 48-array gene expression dataset removed 117 *C. elegans* probes (due to the missing of more than 50% values across arrays). Then the remaining 42,745 probes were collapsed to 28,104 unique genes as multiple probes/probe sets were reduced to one per gene symbol by using maximally expressed probe/probe set measured by average intensity across arrays. Gene expression effects were determined at 1.9, 15.5, 62.5 mg DNAN/L, 187, 750, 3000 mg NTO/L, or 83, 666, 2667 mg NQ/L, in comparison with respective controls. The total number of non-redundant DEGs of the three groups is 2489. Statistical analyses indicated that 2175, 378, and 118 DEGs were affected in NQ-, DNAN-, and NTO-treated nematodes, respectively, with only 11 DEGs shared by all three groups (Fig. 4 and Additional file 1). On the other hand, 60% of DEGs in response to NTO were common with DEGs responding to either NQ or DNAN. Further, 36% of the DEGs in response to DNAN were common with DEGs in response to either NQ or NTO. This result suggests that the three compounds acted on both common and different gene targets.

**Fig. 5:**
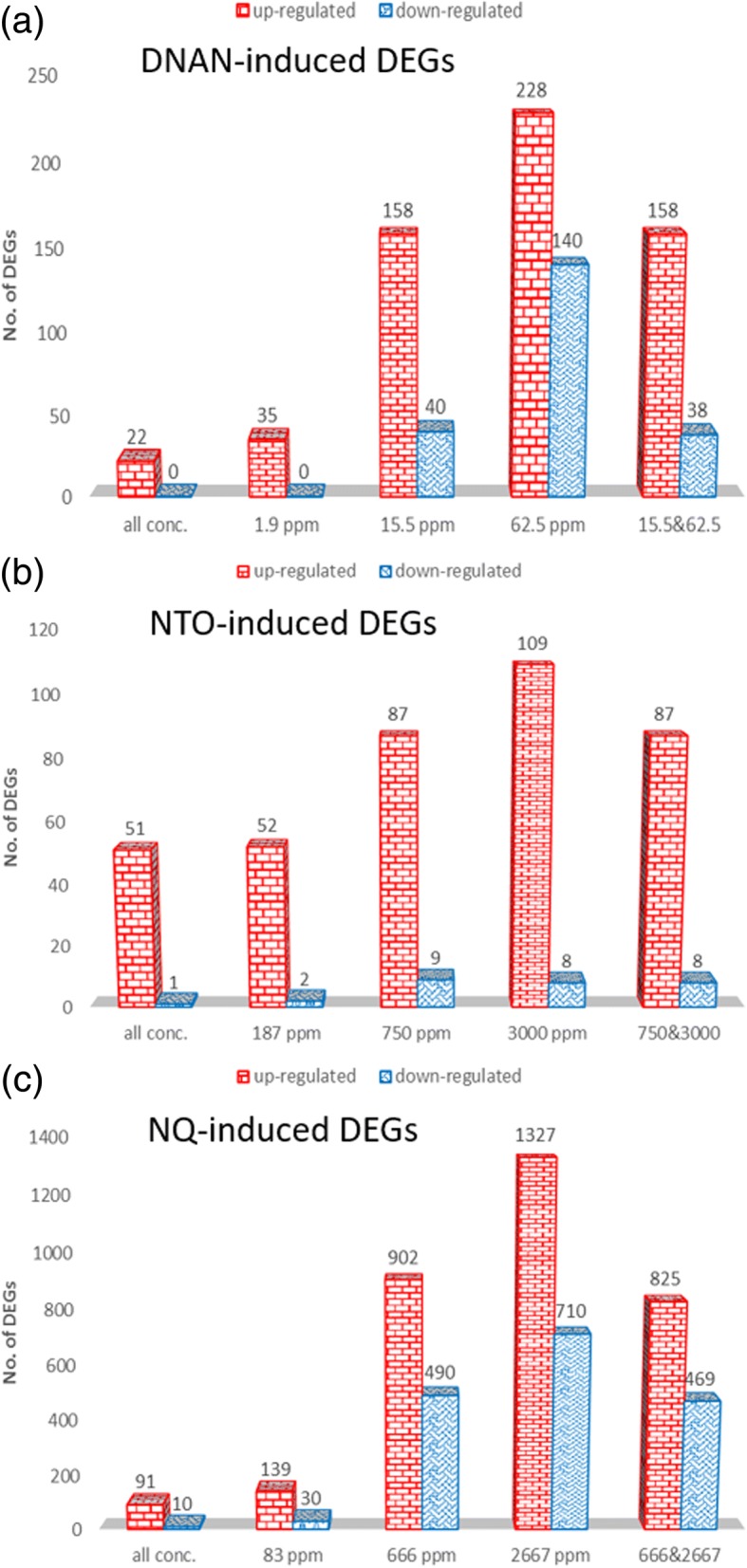
Breakdown of (**a**) DNAN-induced, (**b**) NTO-induced, and (**c**) NQ-induced DEGs by exposure concentration (individual, both medium and high, and all concentrations) and regulation pattern (up- and down-regulated)

A breakdown of the DEGs by exposure concentration and regulation pattern shows that the number of both up- and down-regulated DEGs increased with concentration for all three compounds (Fig. 5). Persistently, more DEGs were up-regulated than down-regulated across all compounds and all concentrations. The proportion of consistently up- or down-regulated DEGs at all three concentrations for each compound was small, i.e., < 6% of the total DEGs for each compound, except that 51 of the 52 DEGs up-regulated at 187 mg NTO/L were also up-regulated at the two higher NTO concentrations. However, DEGs highly overlapped between the two higher concentrations, i.e., 88% to 100% of the DEGs affected at the medium concentration were also affected at the highest concentration.

### Bioinformatics analysis of DEGs and affected Reactome/KEGG pathways

**Fig. 6:**
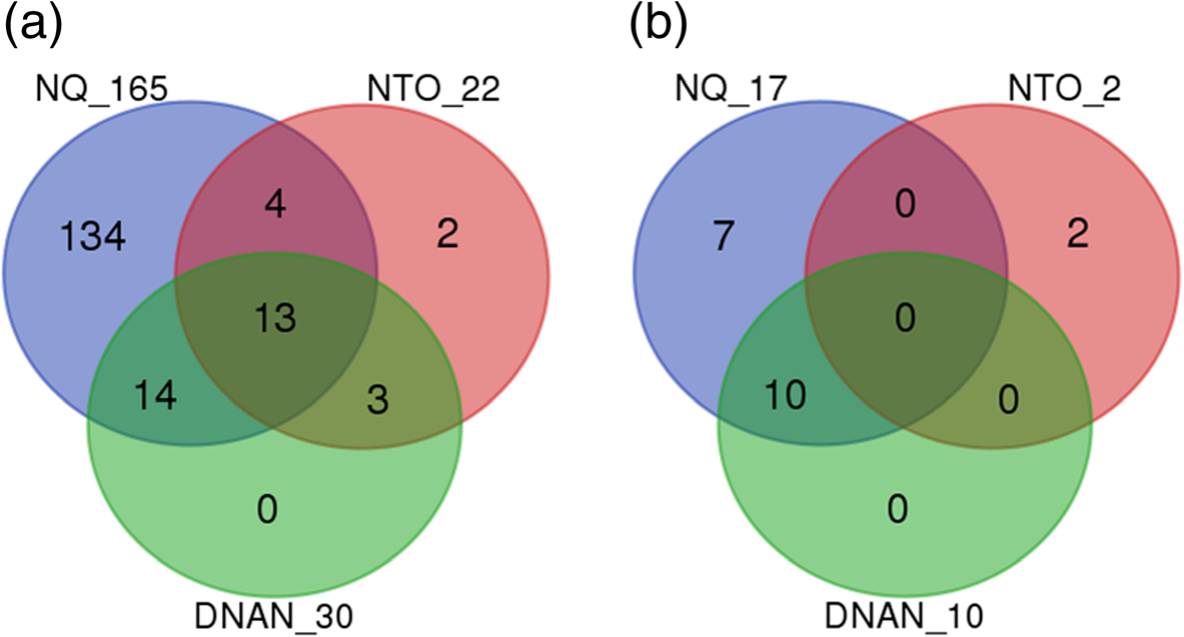
Overlapping of computationally inferred *C. elegans* pathways in the Reactome database mapped by differentially expressed genes (DEGs). **a** All DEG-mapped pathways; **b** Pathways mapped with 4 or more DEGs. See Additional file 3 for the list of all DEG-mapped pathways and DEGs mapped to these pathways

The KEGG gene symbols of the 2489 unique DEGs were first converted to their equivalent UniProt gene identifier using a file linking KEGG Genes to UniProt (Additional file 2), which was downloaded from KEGG LinkDB (http://www.genome.jp/linkdb/). As a result, 1185, 269 and 78 NQ-, DNAN-, and NTO-affected DEGs, respectively, were matched with UniProt gene identifiers (Additional file 2). Then, these UniProt gene identifiers (1404 non-redundant) were mapped to the lowest hierarchical level of *C. elegans*-specific Reactome pathways using a UniProt-to-Pathways mapping file (Additional file 3), which was downloaded from the Reactome pathway database (http://www.reactome.org/pages/download-data/). The following mapping results were obtained: 127 NQ-affected, 30 DNAN-affected, and 14 NTO-affected DEGs were mapped to 165, 30, and 22 Reactome pathways, respectively (Tables 1 and 2; Additional file 3). Up to 15 unique DEGs were mapped to a single pathway (Table 3), and a single DEG was mapped to as many as 10 pathways (Additional file 3). When pathways mapped with 1 to 3 DEGs were removed, there left only 17, 10 and 2 pathways mapped with NQ-, DNAN, and NTO-affected DEGs, respectively (Table 3). The overlapping of all DEG-mapped pathways and those mapped with more than three DEGs are shown in Fig. 6.

**Table 1:** Statistics of differentially expressed genes (DEGs) mapped to the lowest hierarchical level of Reactome pathways: Breakdown of DEGs by the number of pathways that a DEG is mapped to. Blank cells indicate zero DEG or pathway. See Additional file 3 for the detailed listings of mapped DEGs and pathways

Number of pathways/DEG	Number DEGs affected by		
	DNAN	NTO	NQ
1	8	3	59
2	6	9	30
3	1		13
4	1		7
5			3
6			5
7			1
10	14	2	9
Sum	30	14	127

**Table 2:** Statistics of differentially expressed genes (DEGs) mapped to the lowest hierarchical level of Reactome pathways: Breakdown of pathways by the number of DEGs mapped to a single pathway. Blank cells indicate zero DEG or pathway. See Additional file 3 for the detailed listings of mapped DEGs and pathways

Number of DEGs/pathway	Number of pathways affected by		
	DNAN	NTO	NQ
1	17	7	102
2		13	30
3	3		16
4		2	2
5			2
6			3
8			8
9			2
14	9		
15	1		
Sum	30	22	165

**Table 3:** Reactome pathways mapped by four or more DEGs induced by NQ, NTO, or DNAN

Pathway ID	Pathway name	NQ	NTO	DNAN
R-CEL-211979	Eicosanoids		4^a^	
R-CEL-2162123	Synthesis of Prostaglandins (PG) and Thromboxanes (TX)		4	
R-CEL-1660662	Glycosphingolipid metabolism	4		
R-CEL-2046106	Alpha-linolenic acid (ALA) metabolism	4		
R-CEL-193368	Synthesis of bile acids and bile salts via 7alpha-hydroxycholesterol	5		
R-CEL-75105	Fatty Acyl-CoA Biosynthesis	5		
R-CEL-1442490	Collagen degradation	6		
R-CEL-1679131	Trafficking and processing of endosomal toll-like receptor (TLR)	6		
R-CEL-187577	Skp, Cullin, F-box protein containing complex (SCF)-Skp2-mediated degradation of p27/p21	6		
R-CEL-156581	Methylation	8		15
R-CEL-193048	Androgen biosynthesis	8		14
R-CEL-193993	Mineralocorticoid biosynthesis	8		14
R-CEL-194002	Glucocorticoid biosynthesis	8		14
R-CEL-211957	Aromatic amines can be N-hydroxylated or N-dealkylated by CYP1A2	8		14
R-CEL-211981	Xenobiotics	8		14
R-CEL-2142816	Synthesis of (16–20)-hydroxyeicosatetraenoic acids (HETE)	8		14
R-CEL-5423646	Aflatoxin activation and detoxification	8		14
R-CEL-211976	Endogenous sterols	9		14
R-CEL-2142670	Synthesis of epoxy (EET) and dihydroxyeicosatrienoic acids (DHET)	9		14

^a^Number of DEGs mapped to the pathway

**Fig. 7:**
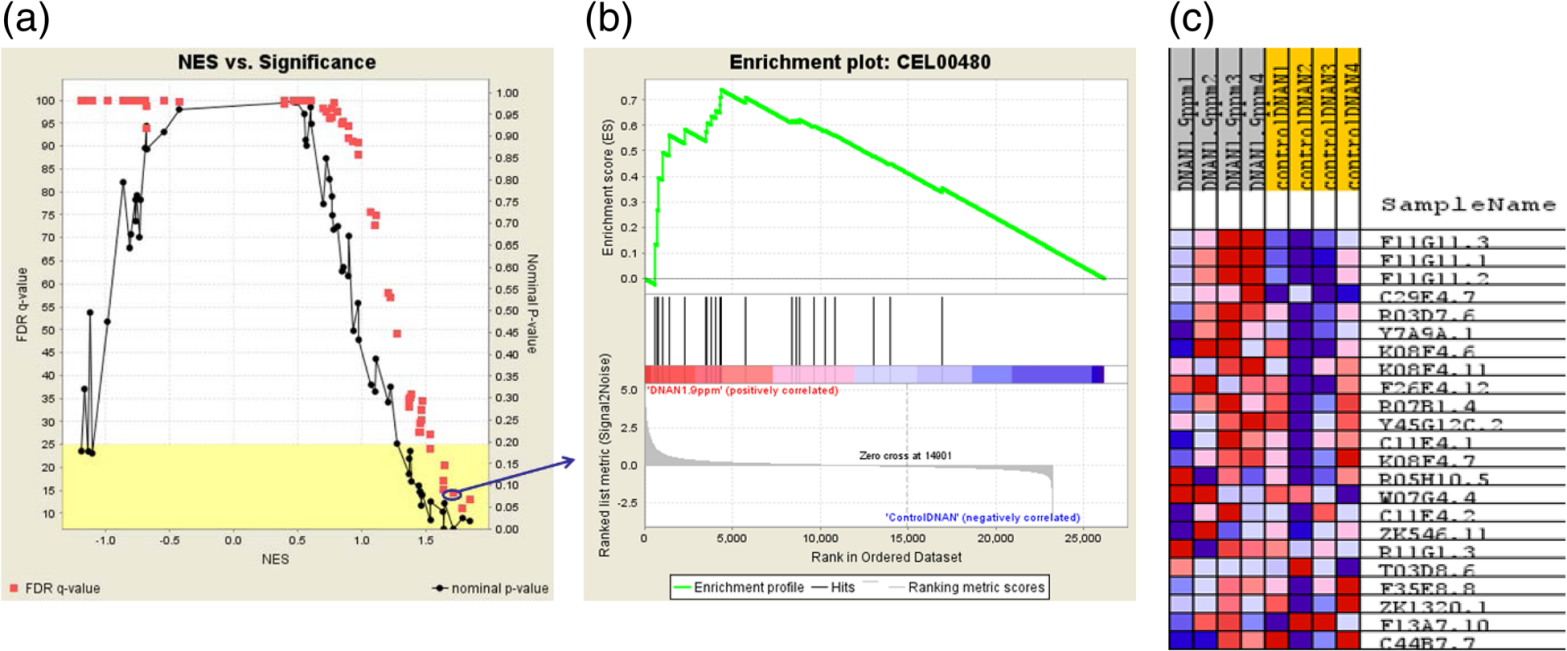
Some examples of gene set enrichment analysis (GSEA) output files: **a** Normalized enrichment score (NES) plotted against false discovery rate (FDR) or nominal *P*-value which were derived from GSEA of 134 gene sets (i.e., KEGG pathways, see Additional file 4) in the Control vs. DNAN_1.9 ppm comparison; **b** Enrich plot of one of the gene set CEL00480 (FDR = 0.14, see the blue circle and arrow in (**a**)) significantly altered by DNAN_1.9 ppm; and **c** Microarray gene expression heat map of the 23 genes mapped to the KEGG pathway CEL00480

To prepare for GSEA, we first mapped nearly 50% (13512) of the probed genes (i.e., genes represented on the microarray) to 134 KEGG-curated *C. elegans* biological pathways (www.kegg.jp) (see Additional file 4 for details). Additional file 4 was uploaded as the gene set file for GSEA analysis together with an expression dataset (relevant sample array data from GSE92365), a phenotype label (e.g., control 1/2/3/4 and DNAN_1.9 ppm 1/2/3/4) file and a chip annotation (i.e., gene symbols in GPL22795) file. GSEA results of these mapped KEGG pathways (see examples of GSEA output files in Fig. 7) revealed that DNAN and NTO affected 12 and 6 pathways, respectively, with 3 in common (Table 4). Although NQ had the highest amount of DEGs, no enriched pathway was statistically significant (False Discovery Rate or FDR < 0.25 and significant at 2 or 3 concentrations).

**Table 4:** Significantly affected KEGG pathways derived using GSEA (FDR < 0.25 at 2 or 3 concentrations)

Pathway ID	Size	DNAN conc.	NTO conc.	KEGG pathway name
CEL00350	15	15.5; 62.5		Tyrosine metabolism
CEL00380	14		750; 3000	Tryptophan metabolism
CEL00480	23	1.9; 15.5; 62.5	187; 750; 3000	Glutathione metabolism
CEL00500	14	15.5; 62.5		Starch and sucrose metabolism
CEL00630	14		750; 3000	Glyoxylate and dicarboxylate metabolism
CEL00830	10	15.5; 62.5		Retinol metabolism
CEL00980	25	15.5; 62.5	187; 750; 3000	Metabolism of xenobiotics by cytochrome P450
CEL00982	27	15.5; 62.5	187; 750; 3000	Drug metabolism - cytochrome P450
CEL00983	17	15.5; 62.5		Drug metabolism - other enzymes
CEL01200	25		750; 3000	Carbon metabolism
CEL02010	12	15.5; 62.5		ATP-binding cassette (ABC) transporters
CEL04070	16	1.9; 15.5		Phosphatidylinositol signaling system
CEL04141	60	1.9; 62.5		Protein processing in endoplasmic reticulum
CEL04310	34	1.9; 62.5		Wnt signaling pathway
CEL04350	24	1.9; 62.5		Transforming growth factor beta (TGFβ) signaling pathway

## Discussion

At exposure concentrations having no significant effects on lethality and reproduction (except for 62.5 mg DNAN/L), we observed significant impacts in acute response to each of the three IM constituents on transcriptome-wide gene expression. Our study provided evidence that transcriptomic effects were concentration-dependent with the lowest exposure concentration significantly altering gene expression, i.e., 1.95 ppm, 187 ppm and 83 ppm for DNAN, NTO and NQ, respectively (Fig. 4). Findings from the comparative toxicogenomic analyses suggest that the three chemicals shared some common target genes and pathways but also acted independently on different genes and pathways (Figs 4, 5 and 6). In the following, we focus our discussion on the altered pathways identified using two different approaches.

**Fig. 8:**
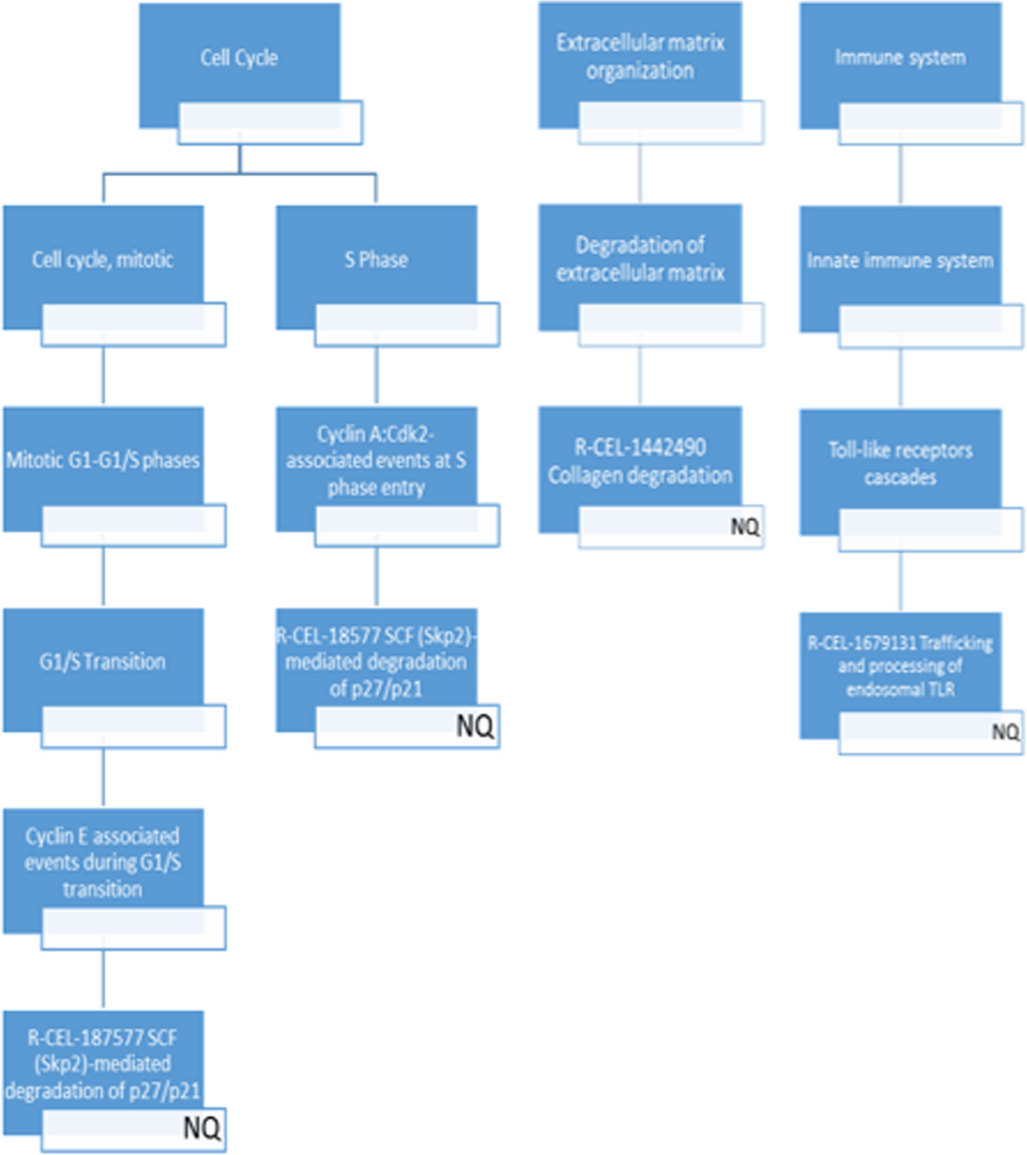
Hierarchical view of DEGs-mapped Reactome pathways (metabolism). Only pathways at the lowest organizational level were used for both mapping and enrichment analyses. The chemical that induced DEGs in the pathway is shown in a white rectangle box at the right bottom corner of a Reactome pathway box. More information about the number of DEGs mapped to each Reactome pathway can be found in Table 3

**Fig. 9:**
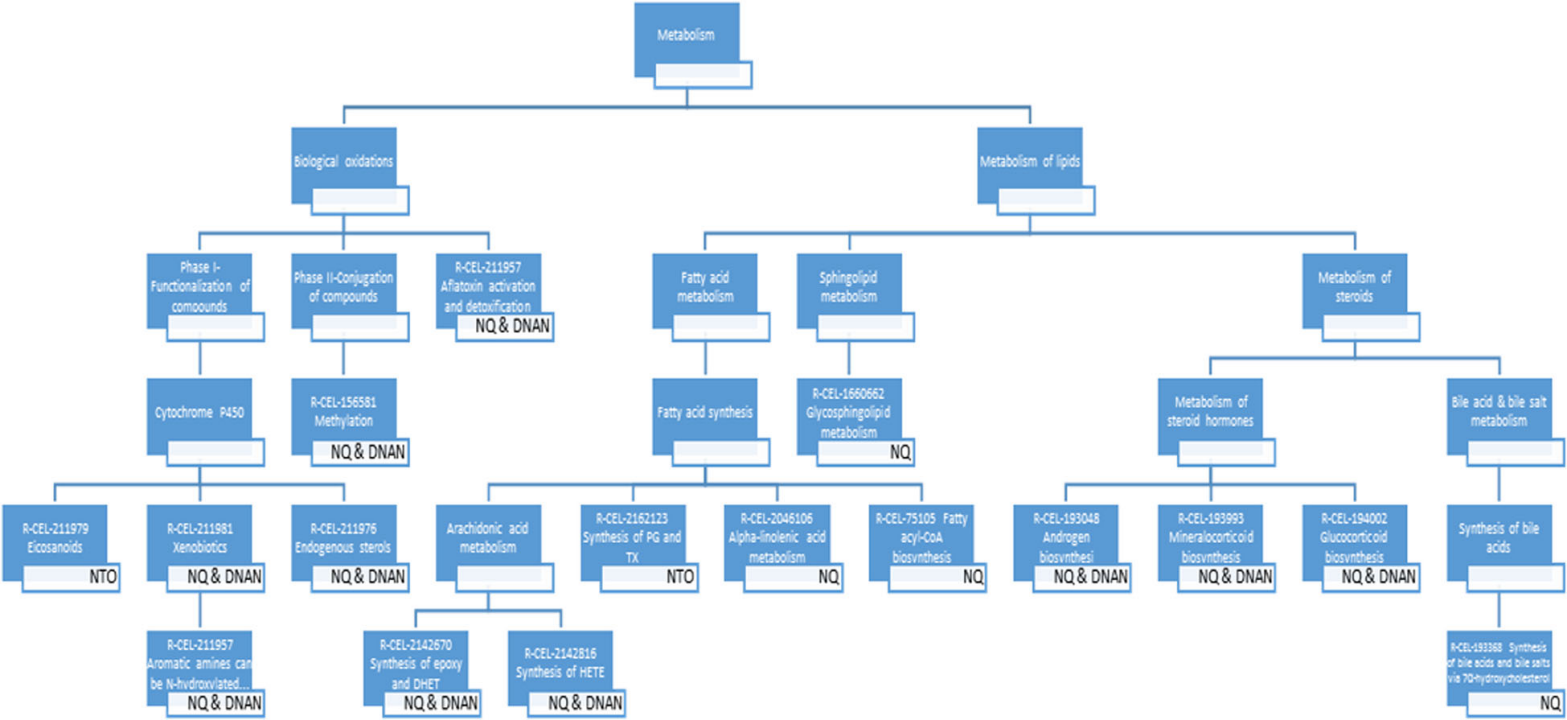
Hierarchical view of DEGs-mapped Reactome pathways (cell cycle, extracellular matrix organization and immune system). Only pathways at the lowest organizational level were used for both mapping and enrichment analyses. The chemical that induced DEGs in the pathway is shown in a white rectangle box at the right bottom corner of a Reactome pathway box. More information about the number of DEGs mapped to each Reactome pathway can be found in Table 3

Our pathway mapping analysis (Table 3 and Figs. 8 and 9) suggests that NQ affected one Reactome pathway (with ≥4 mapped DEGs) each within the cell cycle, extracellular matrix organization and immune system groups, in addition to 12 Reactome metabolism pathways. NTO and DNAN only affected 2 and 10 Reactome metabolism pathways, respectively. All pathways affected by DNAN were also altered by NQ. However, NTO did not share any altered pathways with NQ or DNAN. Interestingly, eicosanoids and synthesis of prostaglandins (PGs) and thromboxanes (TXs), the two pathways affected by NTO, are categorized into cytochrome P450 and fatty acid synthesis, respectively. They are interconnected because PGs and TXs are collectively known as eicosanoids. Specifically, eicosanoids are synthesized primarily from arachidonic acid that is released from membrane phospholipids [44]. Once released, arachidonic acid is acted on by prostaglandin G/H synthases (PTGS, also known as cyclooxygenases (COX)) to form PGs and TXs, by arachidonate lipoxygenases (ALOX) [45]. Altered eicosanoids (PGs and TXs) biosynthesis may lead to aberrant regulation of immunopathological processes ranging from inflammatory responses to cancer and autoimmune disorder [44].

**Fig. 10:**
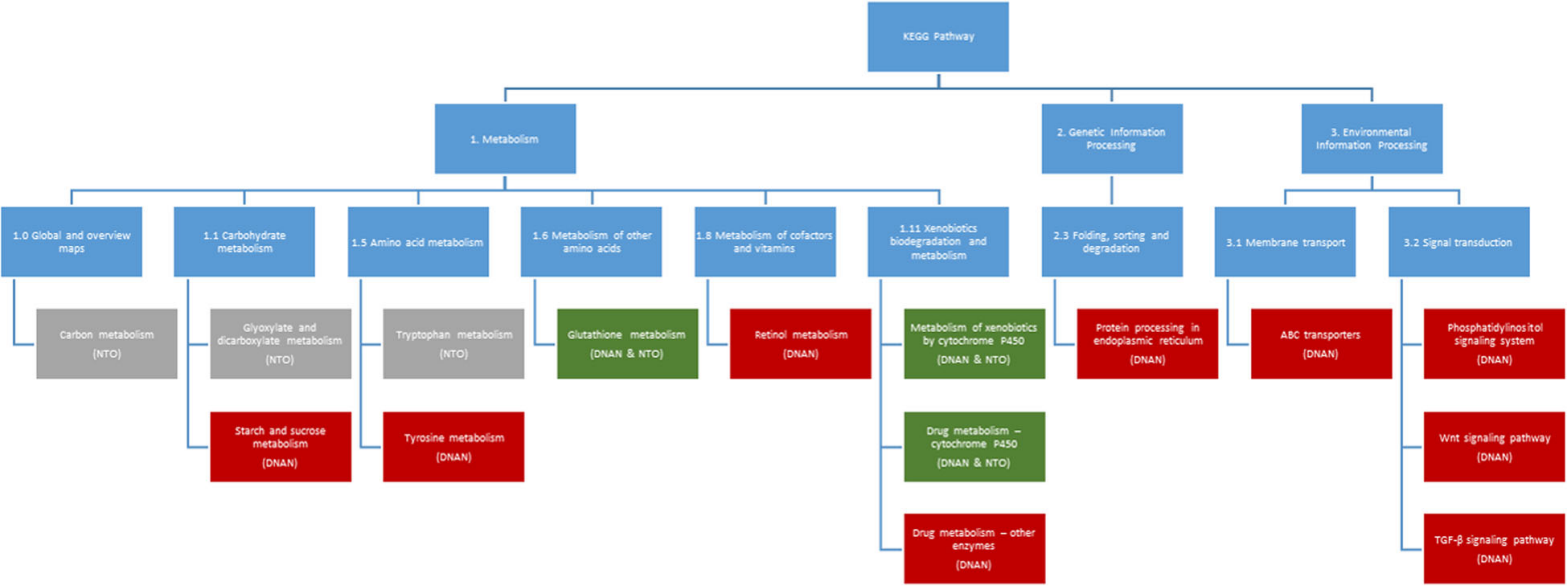
Hierarchical view of GSEA-inferred KEGG pathways enriched with DEGs. Only pathways at the lowest organizational level were used for both mapping and enrichment analyses. The chemical that induced DEGs in the pathway is shown in the parenthesis inside a KEGG pathway box. More information about chemical concentrations significantly altering KEGG pathways can be found in Table 4

Our GSEA results (Table 4 and Fig. 10) indicate that DNAN and NTO both significantly affected KEGG pathways of carbohydrate, amino acid and xenobiotics metabolism, while DNAN also affected genetic and environmental information processing pathways. Interestingly, no pathway was significantly altered by NQ even though NQ-induced DEGs can be mapped to multiple pathways. This was in agreement with the no apical toxicity of NQ up to the highest test concentration (Fig. 3c). It may be hypothesized that NQ does not possess any specific toxicity target but elicit general stress causing adaptive responses in exposed worms.

In contrast to NQ, NTO and DNAN acted on fewer but more specific biomacromolecular targets (Tables 1 and 2), leading to alterations of statistically significant pathways (Table 4). We may hypothesize that NTO and DNAN act independently due to the identified distinct pathways affected by DNAN only, including protein processing, ABC transporter and multiple signal transduction pathways (Table 4 and Fig. 10).

Results from these two analyses should be considered complementary. The differences in the results obtained from the pathway mapping and the GSEA analyses were largely due to the aforementioned differences between the two pathway databases (Reactome vs. KEGG) as well as the different approaches used (mapping vs. statistics). Despite such differences, there also exists commonality between the two analyses, i.e., xenobiotics metabolism by cytochrome P450 (R-CEL-211981 in Table 3 and CEL00980 in Table 4). This suggests that exposure to NQ, DNAN or NTO can all induce the Phase I biotransformation of xenobiotic toxicants mediated by the P450 isozyme system [46].

A primary goal of toxicogenomics research is to identify novel biomarkers and elucidate mode of toxicological action. Findings from our previous earthworm toxicogenomics studies indicate that TNT mainly acts as a reactive oxidative stressor affecting a variety of biological processes such as muscle contraction, fibrinolysis and coagulation, iron homeostasis, and innate immunity [47, 48]. Our further functional assays confirmed chitinase activity and methemoglobinemia-like blood disorder as specific biomarkers in TNT-exposed earthworms [48]. Similarly, RDX was found to primarily cause neurotoxicity and reproductive toxicity by affecting neuronal signaling (cholinergic and GABAergic synapses) pathways and spermatogenesis [49–52]. Electrophysiological assays and reproductive toxicity tests confirmed both neurotoxicity and reproductive toxicity of RDX [49, 52, 53]. Compared with TNT and RDX, we just began a long journey of unveiling the toxicological mechanisms for IM constituents. Although significant transcriptomic effects were observed for all three IM constituents at concentrations that do not cause any lethal or reproductive effects, we ought to carry out more functional assays and measure biochemical, physiological and enzymatic endpoints to discern adaptive and reversible responses or hormesis from permanent and irreversible damages.

A number of ecotoxicological studies have demonstrated that DNAN has a significantly lower toxicity than such legacy explosives as TNT and RDX in such animal models as water fleas and fish [19, 20], earthworms [18], and frog tadpoles [21]. These results suggest that IMs may pose less environmental risks than legacy explosives. This study investigated the parent form of the three IM constituents without considering their possible biotransformation products (e.g., [54–56]). Further studies are warranted to tease apart the contribution of these breakdown products to the adverse effects reflected in the bioassays designed to measure the parent compounds. In addition, toxicogenomic studies need to be conducted for legacy explosives such as TNT and RDX using the same *C. elegans* model in order to better differentiate their modes of toxicological action from those of IM compounds. It should also be noted that microarray-based toxicogenomic studies often serve as preliminary evidence to generate hypotheses for in-depth and confirmative research into toxicological mechanisms. Such research may be conducted using *C. elegans* genetic mutant strains. For instance, Liao et al. [57] employed a chemotaxis assay [58] to identify the neuronal location of olfactory receptors responding to explosives-associated volatiles.

## Conclusions

This comparative toxicogenomics study demonstrated that at individual concentrations not causing significant reduction in nematode reproduction, DNAN, NQ and NTO induced 378, 2175 and 118 DEGs in exposed nematodes, respectively. The three compounds shared both DEGs and DEG-mapped Reactome pathways. GSEA results suggest that DNAN and NTO significantly altered 12 and 6 KEGG pathways, separately, with three pathways in common. NTO mainly affected carbohydrate, amino acid and xenobiotics metabolism, DNAN disrupted protein processing, ABC transporters and several signal transduction pathways, and NQ-induced DEGs were mapped to a wide variety of metabolism, cell cycle, immune system and extracellular matrix organization pathways. Taken together, this study provided supporting evidence that the three chemicals, if mixed (e.g., in IMX-101), may exert independent toxicity by acting on distinct molecular targets and pathways.

## Additional files

Additional file 1: Differentially expressed genes derived for sublethal DNAN, NTO and NQ exposures in *C. elegans*. (XLSX 345 kb)

Additional file 2: Conversion of gene symbols to UniProt identifiers using the KEGG-UniProt linkage file. (XLSX 1258 kb)

Additional file 3: UniProt genes mapping to computationally inferred *C. elegans* pathways in the Reactome database. (XLSX 508 kb)

Additional file 4: Gene set file (i.e., gene mapping to KEGG-curated *C. elegans* pathways) used for gene set enrichment analysis (GSEA). (XLSX 31694 kb)

## Abbreviations

ALOX: Arachidonate lipoxygenases
BC: Blank control
COX: Cyclooxygenases
DEG: Differentially expressed gene
DNAN: 2,4-dinitroanisole
DoD: Department of Defense
DOE: Department of Energy
EC_50_: 50% effective concentration
FDR: False discovery rate
GEO: Gene expression omnibus
GO: Gene ontology
GSEA: Gene set enrichment analysis
IC_50_: 50% inhibitory concentration
IM: Insensitive munition
IMX-101: Insensitive munitions explosive 101
KEGG: Kyoto Encyclopedia of Genes and Genomes
KO: KEGG orthology
LC_50_: 50% lethal concentration
LOAEL: Lowest observable adverse effect level
NES: Normalized enrichment score
NGM: Nematode growth media
NOAEL: Non-observable adverse effect level
NOEC: No observable effect concentration
NQ: Nitroguanidine
NTO: 3-Nitro-1,2,4-triazol-5-one
OD: Optical density
PG: Prostaglandin
PTGS: Prostaglandin G/H synthases
RDX: Research Department formula X
TC: Tissue-culture
TNT: 2,4,6-trinitrotoluene
TX: Thromboxanes
VC: Vehicle control
